# Supplementary material for: Determination of Anti-Xa Inhibitor Plasma Concentrations Using a Universal Edoxaban Calibrator
Source: Diagnostics (Basel). 2023 Jun 20;13(12):2128. doi: 10.3390/diagnostics13122128 (PMC10297422; doi:10.3390/diagnostics13122128)
Supplement: Supplementary file 1 [file diagnostics-13-02128-s001.zip › diagnostics-2376719-supplementary.pdf]

diagnostics

*Supplementary material*

Table S1: Intra- and inter-day (n=6) imprecision (CV) and inaccuracy (bias) for the different analytes (LC-MS/MS)

| Analyte | Concentration (ng/mL) | Interday | | Intraday | |
|---|---|---|---|---|---|
| | | CV (%) | Bias (%) | CV (%) | Bias (%) |
| Apixaban | 40 | 8.3 | 2.8 | 3.7 | 9.9 |
| | 150 | 5.6 | 6.9 | 5.6 | 9.0 |
| | 300 | 8.0 | 8.6 | 7.7 | 7.0 |
| Rivaroxaban | 40 | 6.0 | 7.1 | 3.6 | 7.2 |
| | 150 | 4.4 | 6.1 | 5.1 | 5.9 |
| | 300 | 11.5 | 10.6 | 2.4 | 10.1 |
| Edoxaban | 40 | 5.9 | -9.6 | 4.7 | -8.9 |
| | 150 | 5.2 | -8.7 | 2.6 | -1.5 |
| | 300 | 9.2 | -3.5 | 6.6 | -5.5 |
| Edoxaban M4 | 40 | 6.3 | -3.2 | 3.0 | 3.8 |
| | 150 | 6.6 | -2.9 | 4.1 | 10.8 |
| | 300 | 7.2 | 2.2 | 5.9 | 6.8 |
